# Mitochondrial Retrograde Signalling and Metabolic Alterations in the Tumour Microenvironment

**DOI:** 10.3390/cells8030275

**Published:** 2019-03-22

**Authors:** Dongki Yang, Jaehong Kim

**Affiliations:** 1 Department of Physiology, College of Medicine, Gachon University, Incheon 21999, Korea; dkyang@gachon.ac.kr; 2 Department of Biochemistry, College of Medicine, Gachon University, Incheon 21999, Korea; 3 Department of Health Sciences and Technology, Gachon Advanced Institute for Health Science and Technology, Gachon University, Incheon 21999, Korea

**Keywords:** mitochondria, retrograde signalling, metabolic reprogramming, tumour microenvironment, EMT

## Abstract

This review explores the molecular mechanisms that may be responsible for mitochondrial retrograde signalling related metabolic reprogramming in cancer and host cells in the tumour microenvironment and provides a summary of recent updates with regard to the functional modulation of diverse cells in the tumour microenvironment.

## 1. Introduction

The precise role of the mitochondria in the pathogenesis of specific chronic diseases such as diabetes, neurodegenerative diseases and cancer is still uncertain. Advances in molecular biology and in the field of metabolic research have shown that the metabolic alterations in cancer not only are a simple secondary effect from the aberrant signalling regulation for growth and proliferation but can also act as a primary cause for tumorigenic [1], metastatic [2] and stem cell-like characteristics [3] and can cause therapeutic resistance in cancer [4]. For ATP production, healthy cells commonly use glycolysis in the absence of oxygen and OXPHOS in the presence of oxygen [5]. Despite enhanced aerobic glycolysis (Warburg effect), most cancer cells also maintain mitochondrial respiratory capacity to produce a significant amount of ATP [6,7,8] and functionally competent mitochondria are essential for the survival of cancer cells [9,10,11]. Although cancer cells in general may maintain OXPHOS function, it does not necessarily mean that cancer cells have no defects in mitochondrial respiration. Enhanced glycolysis in certain cancers is clearly due to a functional abnormality of the mitochondria [12,13] from decreased expression of oxidative enzymes and transporters, a truncated TCA cycle, a lowering in the number of mitochondria and defective respiratory chain, a higher sensitivity of mtDNA to oxidative stress such as ROS injury and an increase in natural inhibitors of the mitochondrial ATP synthase [14,15]. Indeed, certain mtDNA mutations compromise ETC functions and result in a shift to aerobic glycolysis, a metabolic phenotype typical for cancer progression. However, dominant OXPHOS, rather than aerobic glycolysis or mixed phenotypes, can also be commonly observed in various types of cancers and is known to be responsible for the metastatic progression of cancer [2,16]. These findings indicate that cancers maintain functional mitochondria, rather than ‘the defective mitochondria’ that Otto Warburg’s colleagues hypothesized and that metabolic flexibility is common in the progression of cancer [2]. The basic components of mitochondrial function, genetics and epigenomic regulation are discussed in detail here [17].

Although cancer research has focused exclusively on cancer cells, the role of stromal and immune cells in cancer progression has become a new centre of focus. Non-transformed stromal, endothelial and immune cells outnumber their neoplastic counterparts in cancer [18,19]. From early carcinogenesis to progression and metastasis, cancer cells interact with various types of stromal cells such as cancer-associated fibroblasts (CAFs), endothelial cells and immune cells in the tumour microenvironment (TME). Indeed, pleiotropic interactions between various cells are responsible for the maintenance and disturbance of homeostasis in the TME [20]. Cancer-associated metabolic changes, including metabolic flexibility, are not a strictly uniform feature of malignant cells. They also differ across distinct cancers and are found even in non-transformed cells in the TME [21,22], indicating that metabolic flexibility can occur not only from genetic changes in genomic nDNA of cancer cells but also from modulation of metabolism by cells in the TME depending on the requirements of these cells to adapt. Since rapid cell proliferation requires accelerated production of the basic cellular building blocks for assembling new cells, differences in metabolism between cancer cells and non-transformed stromal and endothelial cells together can fuel cancer growth by lactate shuttling, maximally producing substrates for biosynthesis [23,24,25]. However, the mechanism responsible for the pleiotropic metabolic flexibility observed in various cells in the TME remains unclear. It has been speculated that mitochondria retrograde signalling can be responsible for the metabolic flexibility and progression of cancer [26,27,28].

**Figure 1:**
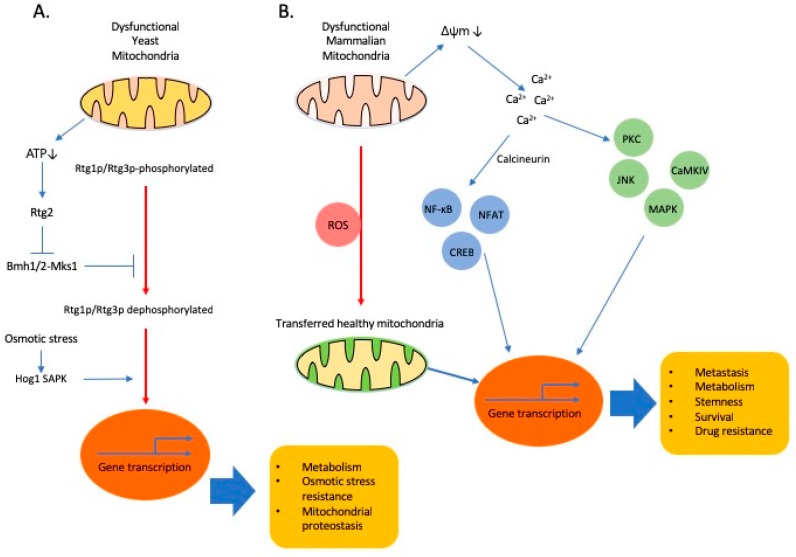
Retrograde signalling and cellular adaptations. (**A**). In yeast, Rtg dependent signalling is the first found major pathway through which mitochondria communicates with nucleus. Rtg1p and Rtg3p are basic helix-loop-helix/leucine zipper (bHLH/LeuZip) transcription factors and nuclear translocation of Rtg1/3p is dependent on partial dephosphorylation of Rtg3p. Rtg2 promotes the dephosphorylation of Rtg3p and its nuclear translocation [37]. Osmotic stress activates Hog1 SAPK. Hog1 SAPK binds to the Rtg1/3p transcription factor and allows its translocation to the nucleus [38]. (**B**). Mitochondrial dysfunction, such as mitochondrial DNA (mtDNA) depletion or OXPHOS inhibition, triggers mitochondrial retrograde signalling in mammalian cells. For example, mitochondrial stress can activate a Ca^2+^-dependent retrograde signalling that is comprised of two branches: one mediated by calcineurin for the nuclear translocation of NF-κB or CREB or NFAT and the other directly dependent on activation of Ca^2+^-dependent protein kinases, such as PKC, JNK, MAPK and CAMKIV. Horizontal transfer of healthy mitochondria from stromal cells in the TME can restore mitochondrial functions and is suggested to participate in the retrograde signalling. Emerging evidences show that ROS stimulates horizontal transfer of mitochondria [39,40].

The significance of mitochondria in the regulation of metabolism is reflected by their involvement in multiple signalling pathways. Altered energy metabolism with a diverse range of metabolic profiles is commonly observed in cancer cells [26], involving genetic alterations not only in nDNA but also in mtDNA and changes in mtDNA copy number, a phenotype recently speculated to originate from the ‘mitochondria to nucleus crosstalk.’ Mitochondrial retrograde signalling is a major form of mitochondria to nucleus crosstalk, which enables extensive communication between the mitochondria and the nucleus, influencing many cellular and cancer phenotypes including changes in metabolism, stemness, survival, drug resistance and metastasis. Mitochondrial retrograde response in response to environmental clues was discovered in S. cerevisiae [29], a direct mitochondrial retrograde response pathway was first described in response to mtDNA depletion in S. cerevisiae [30] and elegant studies established that the retrograde signalling is conserved in yeast and mammals [31,32,33,34]. In yeast, Rtg1p and Rtg3p are transcription factors, forming a dimer that translocates from the cytosol to the nucleus to regulate gene expression, while Rtg2p functions as a sensor of mitochondrial stress (Figure 1) [35]. Although mammalian orthologs of these proteins have not been found [36], similar signalling pathways, to be discussed in the next section, definitely function in mammals.

This review explores the molecular mechanisms responsible for metabolic reprogramming related to mitochondrial retrograde signalling and provides a summary of recent updates regarding the functional modulation of cancer and host cells in the TME by mitochondrial retrograde signalling.

## 2. Mitochondria to Nucleus Crosstalk: Mitochondria Regulates Nuclear Events

Due to the pivotal regulatory function of the mitochondria in homeostasis, cells have regulatory systems to maintain the integrity, mass and metabolic functions of the mitochondria. The majority (1500–2000) of mitochondrial proteins are encoded by the nuclear genome, so communication among cytoplasmic, nuclear and mitochondrial compartments is essential for maintaining mitochondrial function and cellular homeostasis [41,42]. The two main signalling systems that link the mitochondria and the nucleus are nucleus to mitochondria anterograde signalling and mitochondria to nucleus retrograde signalling, which comprise bi-directional communication between the mitochondria and the nucleus. Through control of transcription and translation of genes, which directly regulate mitochondrial biogenesis, nuclear signalling to the mitochondria (anterograde signalling) regulates OXPHOS and mitochondrial biogenesis in response to environmental signals received by the nucleus [43].

Mitochondria endlessly and extensively crosstalk with the nucleus and the cytosol, activating all of transcriptional, translational and post-translational processes aimed at restoring proper mitochondrial function. Mitochondrial retrograde signalling, an emerging area of mitochondrial research, comprises signalling pathways by which dysfunctional mitochondria communicate with the nuclear genetic compartment to relay metabolic, oxidative and respiratory stressful conditions prevailing in the mitochondria as cellular adaptation [37,41]. Retrograde signalling is very pleiotropic with respect to its origin, functional mediators involved and the resulting phenotypes. Since it is a cellular adaptation process, retrograde signalling does not necessarily result in full restoration of mitochondrial homeostasis.

Mitochondrial dysfunction can be induced by various stress factors including low mtDNA copy number, mtDNA mutations, nDNA mutations that affect mitochondrial function, mitochondrial respiratory defects that result in changes in the mtROS levels and functional competence of respiratory chain complexes. Unfortunately, we do not have clear understanding about the identity of specific signalling molecules that can trigger the retrograde signalling so far either in yeast or mammals. Mitochondrial dysfunction can affect a complex cytosolic and mitochondrial network of protein homeostasis pathways, found and mostly studied in yeast or C. elegans [36,44,45]. By inhibiting protein synthesis and by activation of the proteasome, unfolded protein response activated by mistargeting of proteins (UPRam) is beneficial for the cells, providing a means for buffering the consequences of physiological slowdown in mitochondrial protein import and for counteracting pathologies that are caused or contributed by mitochondrial dysfunction [46]. Mitochondrial unfolded protein response (UPRmt) is a transcriptional response to increase mitochondrial localized molecular chaperones and proteases to promote the recovery of mitochondrial proteostasis [47]. UPR mt also results in a reduction of nuclear and mtDNA-encoded OXPHOS transcripts to reduce the substrate burden on the overwhelmed proteostasis in stressed mitochondria. In C. elegans, ATFS-1, a transcriptional factor participating in retrograde signalling, was found to limit the accumulation of OXPHOS transcripts during mitochondrial stress and also to stimulate OXPHOS recovery by matching the expression of OXPHOS genes to the proteostatic capacity of mitochondria [48]. However, to our knowledge, it is currently unknown whether direct regulators of retrograde signalling other than G-Protein pathway suppressor 2 (GPS2), yet to be identified, exist in mammals [49].

From studies regarding metabolic diseases, inflammation and cancer, we have a lengthy list of key small molecules, participating in retrograde signalling, including but not limited to, ROS [50,51], NAD+/NADH ratio [52,53], acetyl-CoA [54,55], ATP [56], Ca^2+^ [57,58] and oncometabolites [59,60]. Depending on cell type and conditions, there are essentially two branches in Ca^2+^ mediated retrograde signalling pathway: (1) Ca^2+^/calcineurin-mediated retrograde signalling for the nuclear translocations of transcription factors, NF-κB [61], NFAT, CREB and HnRNPA2 [41,62,63]; and (2) direct activation of Ca^2+^-dependent protein kinases, such as PKC, JNK, MAPK and CAMKIV [61,64]. Activation of these signalling pathways in epithelial cells converges on the upregulation of genes affecting several cellular functions, including apoptosis resistance, multi-drug resistance, invasion and EMT (Figure 1). Oncometabolites are metabolites whose abundance is significantly increased in cancer cells compared to normal cells. Increasing evidence shows that oncometabolites contribute to cancer progression. In addition, we advise our readers to refer to several excellent articles with more detailed information about retrograde signalling involved in epigenetic changes [37,41,65] and posttranslational modifications of proteins including c-Src, MAPK, AMPK, PARPs, SIRT1 [26,66,67], in diverse pathological conditions.

Considerable attention is given to these mitochondrial stresses because they drive both beneficial and pathogenic adaptive responses [68]. Retrograde signalling appears to be capable of affecting a wide range of processes in cancer progression including activating signalling pathways that regulate metabolic adaptation, antioxidant systems, cellular proliferation, apoptosis-resistance, chemo-resistance and cellular migration and invasion [69,70]. Emerging evidence suggests that it is responsible for metabolic flexibility observed between different cancers and even between cancer cells in the same cancer tissue. Indeed, mitochondria-to-nucleus retrograde signalling in cancer may be a highly plausible mechanism by which altered mitochondrial function modulates adaptive changes in nuclear gene expression and metabolism mediated by specific transcription factors [62,64] towards enhanced tumorigenesis and invasiveness. In addition, we are beginning to gather seemingly still fragmentary but highly important evidence suggesting that mitochondria can regulate several important nuclear events including genetic and epigenetic changes in cancer cells [17,41,71].

Reduced mtDNA content has been associated with aggressive features including a metabolic shift to glycolysis, apoptosis and increased invasiveness in multiple cancer types, such as prostate [72,73,74] and colorectal [75] cancers. However, caution must be taken in interpreting these findings because we do not have enough convincing data to show that retrograde signalling actually engages in the whole stages of cancer progression. Cancer cell proliferation in primary sites, intravasation, survival, migration in blood vessels, extravasation and colonization of tumour cells in distant sites are distinct steps in cancer progression. It has been suggested that retrograde signalling actually counts in the late stage of gene expression reprogramming that alters the metabolic phenotype during malignant transformation [76]. Mitochondrial dysfunction induced by mtDNA depletion promotes EMT in breast epithelial cells through a Ca^2+^/calcineurin-mediated mitochondrial retrograde signalling that triggers transcriptional activation of SLUG, SNAIL and TWIST, MMP-9 and the mesenchymal markers, vimentin, fibronectin and N-cadherin, with a corresponding decrease in the epithelial marker, E-cadherin [77]. Of note, reduced mtDNA content has been directly correlated with induction of EMT through activation of mitochondria-to-nucleus signalling [27,70] and revitalization of OXPHOS is commonly observed in cancer cells undergoing EMT [16,78,79,80,81]. The changes in mitochondrial genome are likely metastasis modifiers rather than drivers [28].

There is accumulating evidence that mtDNA can be transferred between cells or species [79,80,82,83,84]. Mitochondria can move from one cell to another by various intercellular structures such as tunnelling nanotubes (TNTs) or cytoplasmic bridges [84,85]. Mesenchymal stem cells (MSCs) sense mitochondria released from damaged cells as danger signals to activate their rescue properties for the damaged cells [86]. Foreign mitochondria from damaged cells were engulfed and degraded by MSC, leading to induction of the cytoprotective enzyme heme oxygenase-1 and stimulation of mitochondrial biogenesis in MSC. As a result, the rescue capacity of MSC to transfer their mitochondria to injured cells to combat oxidative stress injury was enhanced. Horizontal transfer of mtDNA from the host cells in the TME to cancer cells with compromised respiratory function to re-establish respiration and metastatic efficacy was also recently shown [80,84,87]. Transfer of mitochondria between leukemic cells and bone marrow mesenchymal stem cells is increased by chemotherapy [39]. It has been shown that uptake of mitochondria by leukemic cells increases oxidative phosphorylation and favours survival, indicating new insights into a novel mechanism of drug resistance [88,89]. Another report also showed that cancer cells uptaking mitochondria displayed chemoresistance, indicating functional aspects of mitochondrial acquisition beyond respiration recovery [90]. It may be feasible that interfering with transfer of mtDNA together with targeting OXPHOS metabolism would be an efficient adjuvant strategy to affect the intrinsic crucial metabolic function of cancer cells and the supportive function of TME. Some of the outstanding questions would be (1) what are mechanisms that are responsible for the transfer of host mtDNA to cancer cells in TME? (2) can mtDNA from dysfunctional mitochondria in cancer cells move to the host cells in turn? (3) would the metabolic profiles of the host cells in the TME be affected from the mtDNA transferred from cancer cells? and (4) does the transfer of mtDNA extend the spectrum of retrograde signalling as communication between different cells?

## 3. Oncometabolites from Dysfunctional Mitochondria

Increasing evidence shows that oncometabolites, participating in the retrograde signalling, contribute to cancer progression. For now, the list of established oncometabolites is very short and consists of 2-hydroxyglutarate (2HG), succinate and fumarate, that result from oncogenic mutations in isocitrate dehydrogenase (IDH), succinate dehydrogenase (SDH) or fumarate hydratase (FH), respectively [60]. Since their function is often regulated by both their substrate and the product, metabolic enzymes can directly sense the supply and demand of nutrients. Thus, the capability of metabolic enzymes to both sense metabolic stress and also regulate nuclear gene transcription is an efficient way by which an adaptive response can be achieved. A major functional mechanism of 2HG is their structural similarity to α-KG, which allows 2HG to act as a competitive inhibitor of α-KG-dependent enzymes including the Jumonji-C domain containing histone demethylase (JMJD/JHDM) and the 10-11-translocation methylcytosine dioxygenase (TET) families of chromatin-modifying enzymes and the prolyl hydroxylases (PHD) family [91]. The TCA cycle intermediate, α-KG, is a co-substrate for many enzymes in the cytoplasm and the nucleus, including JMJD/JHDM and TET families of chromatin-modifying enzymes and the PHD family. Glutamine-derived α-KG contributes to TET-dependent demethylation reactions [92]. Mutant versions of cytoplasmic and mitochondrial IDH isoforms, IDH1 and IDH2, respectively, reduce α-KG to generate 2HG [93]. Therefore, through the production of oncometabolite 2HG, mitochondria exert a strong influence on chromatin structure associated with DNA hypermethylation and cause a broad epigenetic change to promote cancer progression [59,94]. 2HG also inhibits the enzymatic activity of cytochrome c oxidase and ATP synthase [70,95] and alters the gene expression of TCA cycle enzymes in cancer cells [96]. These findings suggest that the accumulation of 2HG contributes to the changes in energy metabolism in IDH-mutant cancer cells.

Succinate and fumarate accumulation resulting from mutations in SDH and FH, respectively, stabilize HIF-1α via PHD inhibition, reinforcing the Warburg effect [91,97]. Accumulation of fumarate and succinate also inhibits the α-KG dependent histone and DNA methylases, the JMJD/JHDM and TET family of proteins, respectively [98,99,100,101]. The accumulation of intracellular fumarate can result in the succination modification of cysteine residues within the Keap1 and results in an increase in levels of NRF2 from abrogation of Keap1-mediated degradation of NRF2 [102]. NRF2, acetylated by transcription coactivator and acetyltransferase, p300, activates several antioxidant genes and supports cancer formation. Decreased level of FH can increase the levels of both NRF1 and NRF2 [103] and NRF1 also promotes mesenchymal transition and spheroid survival in mammary epithelial cells by stimulating OXPHOS [81]. Activating mutations of NRF2 or treatment of cancer cells with antioxidants can not only reduce level of ROS but also turn on oncogenic activities [104]. Additionally, the accumulation of fumarate might promote tumorigenesis by inhibiting α-KG-dependent genome-wide histones and DNA methylations, resulting in epigenetic alterations in gene expression [101] or by increasing ROS dependent signalling via generation of succinated glutathione [105]. Fumarate can increase histone H3 methylation by inhibiting KDM2B demethylase and promote binding of DNA-dependent protein kinase and the recruitment of end-processing enzymes for DNA repair [101,106,107]. In conclusion, mutations in mitochondrial metabolic enzymes, IDH2, SDH or FH, results in abundant oncometabolites and leads to epigenetic changes, further dysfunction of mitochondria, production of ROS, cancer progression and notably, increased EMT. Table 1 summarizes functions and producers of oncometabolites.

## 4. Nuclear Metabolic Enzymes as New Regulators of Retrograde Signalling

**Figure 2:**
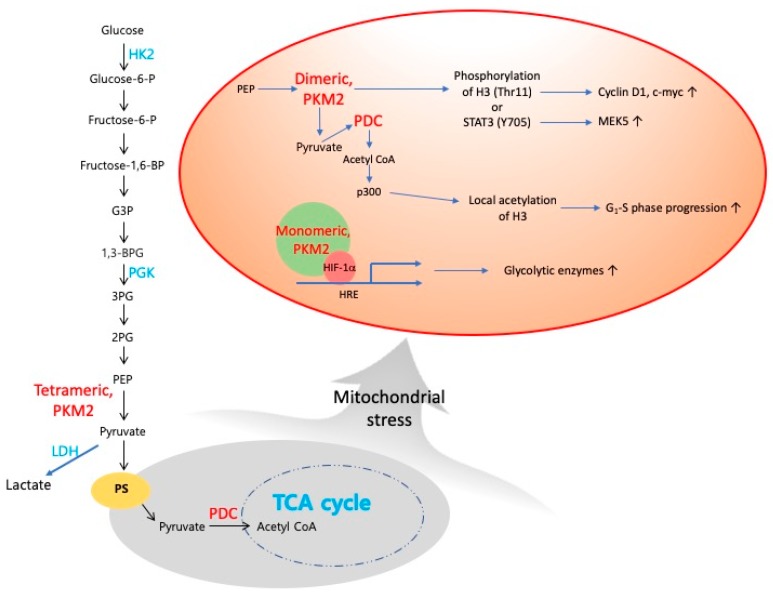
Translocation of PKM2 or PDC into nucleus, where it serves as transcriptional coactivator and as a protein kinase or as a producer of acetyl CoA to modulate transcriptional program. In nucleus, dimeric PKM2 becomes a protein kinase using PEP as a phosphate donor. PKM2 is able to phosphorylate STAT3 at Y705 and promotes transcription of MEK5. PKM2 directly binds to and phosphorylates histone H3 at threonine 11 upon epidermal growth factor (EGF) receptor activation [109]. PKM2 can function as a transcriptional coactivator, promoting HIF-1α binding activity to DNA and thereby participating in a positive feedback loop with HIF-1α [114]. Grey circle denotes mitochondrial events and orange circle indicates nuclear events. Subcellular localization of glycolytic metabolites and tetrameric PKM2 is in cytoplasm. PKM2 and PDC are shown in red. Canonical cytosolic functions of HK2, LDH and PGK are shown here in blue. Abbreviations: G3P, glyceraldehyde 3-phosphate; 3PG, 3-phosphoglyceraste; 2PG, 2-phosphoglyceraste; PEP, phosphoenolpyruvate; PDC, pyruvate dehydrogenase complex; PS, pyruvate symporter.

Recent updates indicate that many metabolic enzymes found in the cytosol or mitochondria can move to the nucleus and have non-canonical functions [71,108,109,110,111], directly linking metabolism with gene transcription, particularly epigenetic mechanisms such as methylation of DNA and histones or acetylation of histones. These findings suggest that metabolic enzymes themselves can participate in the retrograde signalling from their non-canonical function in the nucleus. Both cytoplasmic pyruvate kinase (PKM2) and mitochondrial pyruvate dehydrogenase complex (PDC) can translocate to the nucleus and form a complex with p300, which locally produces acetyl-CoA to acetylate histones and can facilitate locally confined specific gene transcription [111,112]. Nuclear PKM2 produces pyruvate from phosphoenolpyruvate (PEP), which is used by PDC for the production of acetyl-CoA, which in turn phosphorylates histone H3 [110] and STAT3 [109], as a novel non-canonical kinase using PEP instead of ATP as the phosphate donor. Like acetylation, phosphorylation of H3 is also an important histone modification, which promotes cell-cycle progression and cancer growth by increasing the expression of cyclin D1 and c-Myc [110]. Independent of its kinase activity, PKM2 can function as a co-transcription factor, promoting HIF-1α binding activity to DNA and thereby participating in a positive feedback loop with HIF-1α, which upregulates several glycolytic enzymes including PKM2 itself [113,114]. Nuclear PDC levels are increased in a cell-cycle-dependent manner and in response to serum or mitochondrial stress, with a concomitant decrease in mitochondrial PDC levels, suggesting a translocation of PDC from the mitochondria to the nucleus [112]. Indeed, whole-transcriptome analysis revealed that mitochondria have the ability to regulate the expression of more than 66% of genes within the human genome, including the epigenetic/chromatin remodelling machineries [115,116]. Acetyl-CoA can influence posttranslational acetylation on histone tails and change the nuclear epigenome both globally throughout the nucleus and locally for specific histones and proteins. Translocation of mitochondrial PDC to the nucleus promotes acetyl-CoA synthesis in the nucleus, which is required for histone acetylation and epigenetic regulation [112]. Nuclear PDC increases acetylation of specific lysine residues on histones, upregulates expression of phosphorylated Rb, E2F, Cdk2 and cyclin A and promotes G1-S phase progression and expression of S phase markers [112,117]. Mitochondrial stress activates Akt1 and Akt1 mediates transcription activation via phosphorylation and activation of p300 [118]. Acetylation of PKM2 by p300 promotes its nuclear translocation and its non-canonical function as a transcription regulator and a kinase, respectively [119]. Acetylation of the c-Myc oncoprotein by p300 increases its stability and the transcription of its target genes [120], also suggesting a link between mitochondrial stress, acetylation events in the nucleus and cancer progression. Of note, mitochondrial stress can promote nuclear translocation of PDC [112], in addition to the nuclear translocation of cytosolic PKM2, indicating that nuclear translocation of mitochondrial enzyme can be triggered by mitochondrial dysfunction. Our Figure 2 shows a functional summary of nuclear PKM2 and PDC.

Other glycolytic enzymes such as hexokinase 2 (HK2), lactate dehydrogenase (LDH) and 3-phosphoglycerate kinase (PGK) can also move to the nucleus and perform their non-canonical functions. HK2 is an enzyme catalysing the first committed step of glycolysis and found overexpressed in many cancer cells. In the nucleus of HeLa cells, HK2 is found in nucleus and low glucose environment or Akt inhibition augments their nuclear localization [121,122]. In yeast, HK2 regulates its incorporation into the repressor complex of the Mig1-dependent gene promoters in response to cytoplasmic glucose level, indicating its role as a fuel sensor regulating expression of other metabolic enzymes [123]. Y238 phosphorylation of LDH is important for the nuclear translocation, although upstream molecule responsible for the phosphorylation is not clear [124,125]. Nuclear LDH is increased under oxidative stress [126] and binds DNA, stimulates UV-induced DNA repair [127]. Nuclear LDH is also increased by E7-induced ROS accumulation in cervical cancer cells, performs a non-canonical enzyme activity to produce α-hydroxybutyrate and triggers DOT1L mediated histone H3K79 hypermethylation, resulting in the activation of antioxidant responses and Wnt signalling pathway in cervical cancer cells [128]. PGK stimulates DNA synthesis catalysed by DNA polymerase α and ε on single-stranded DNA [127]. LDH in nucleus can also interact with SIRT1 and regulate epigenetic modifications by manipulating NAD+, indicating an intricate link between metabolism and the processing of genetic information [126].

We anticipate that further studies on the identification of metabolic triggers that enable nuclear translocation of metabolic enzymes will reveal that specific nuclear metabolic enzymes are potent participants of the retrograde signalling.

## 5. Retrograde Signalling and Metabolic Switching in the Tumour Microenvironment

Cancer-related non-resolving inflammation in the TME is a hallmark of cancer. TME is generally hypoxic, ROS rich and an acidic environment. Recent findings clearly show that both, a shift to Warburg effect or OXPHOS or combined metabolic phenotypes exist in rapidly proliferating cells, including various types of immune cells, most notably in macrophages and T cells and determine the function of the immune cell subsets in disease conditions such as those in inflamed tissue, obese adipose tissue or cancer [129,130,131,132]. For example, due to metabolic needs to maintain a higher level of ATP, tolerogenic dendritic cells show the highest OXPHOS activity and production of ROS, increased spare respiratory capacity and more pronounced glycolytic capacity and reserve compared to immunogenic mature dendritic cells [133].

**Figure 3:**
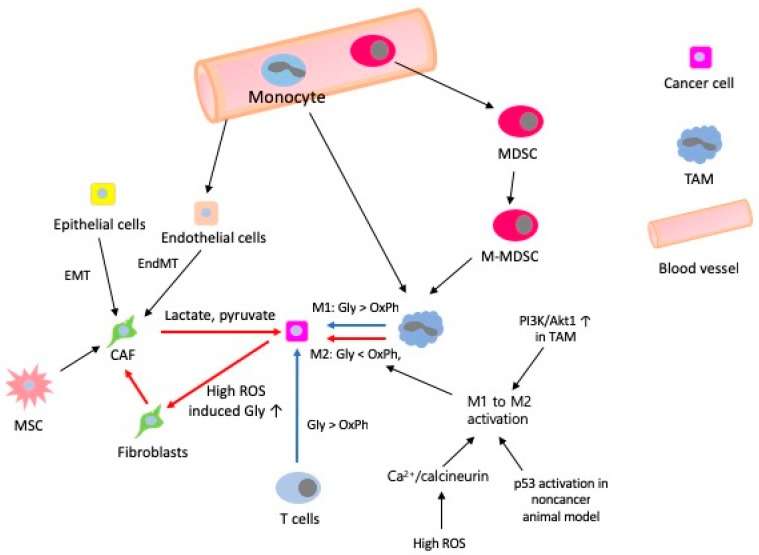
Summary of metabolic switching in the TME. Red arrows indicate immune suppressive response favourable to growth and survival of cancer cells. Blue arrows indicate immune stimulatory response detrimental to cancer cells. Abbreviations: EndMT, endothelial to mesenchymal transition; Gly, aerobic glycolysis; MDSC, myeloid derived suppressor cell; M-MDSC, monocytic myeloid derived suppressor cell; OxPh, oxidative phosphorylation; TAM, tumour-associated macrophage.

M1-associated inhibition of mitochondrial OXPHOS is the factor responsible for preventing M1-like to M2-like activation [134] and glycolytic stimulation is not required for M2-like activation when OXPHOS is intact [135], indicating that metabolism determines macrophage activation. M2-like activated macrophages exploit FAO to fuel OXPHOS, rather than aerobic glycolysis that M1-like proinflammatory activated macrophages exploit for ATP production [136,137,138]. The reports that inhibition of ROS from ETC with metformin [139] or activation of AMPK with AICAR promotes M1 like to M2 like activation [140] indicate that boosting FAO or OXPHOS can be suggested to switch macrophage class from M1-like to M2-like [141]. A “shift” from OXPHOS to aerobic glycolysis is a hallmark of T cell activation [130]. T cells, if not activated, show low levels of metabolic requirements, use OXPHOS to maximize production of ATP as an energy source and engage scarcely in biosynthesis, whereas activated T cells use aerobic glycolysis to produce effector molecules for rapid cellular proliferation [138]. Mitochondrial oxidative metabolism supports immunosuppression and lineage commitment of Tregs [142,143,144]. Cancer aggressiveness is promoted by metabolic synergy between cancer cells and stromal cells [145]. Metabolic synergy induces efficient utilization of catabolites by cancer cells in TME [24] and cancer cells induce aerobic glycolysis in neighbouring fibroblasts by providing a hypoxic ROS-rich microenvironment [24,146]. Induced fibroblasts differentiate to myofibroblasts and upregulate MCT4 to secrete lactate and pyruvate that transform the normal stroma to ultimately help cancer cells grow (the Reverse Warburg effect). All these findings indicate that cells in the TME show mixed metabolic phenotypes of aerobic glycolysis and OXPHOS, resulting from various interactions between different cells or metabolic stress (Figure 3).

Therefore, with metabolic regulation via suppression of aerobic glycolysis and concomitant promotion of OXPHOS, would it be possible that mitochondrial retrograde signalling, an adaptive mechanism to restore mitochondrial homeostasis in response to mitochondrial stress, can also participate in activation of immune cells? If yes, is it responsible for the metabolic flexibility in the TME? It appears that we have some answers suggesting that retrograde signalling can suppress glycolysis and promote OXPHOS in myeloid cells. Mitochondrial ROS is important both for M1-like and M2-like activation [147,148] and can induce retrograde signalling by alterations in mitochondrial membrane potential (Δψm) and activation of Ca^2+^/calcineurin dependent factors, suggesting an involvement of the retrograde signalling pathway in M1-like to M2-like activation. Indeed, in murine macrophages, mitochondrial dysfunction induced by hypoxic insult or ATP synthase inhibitor, activates Ca^2+^/calcineurin or mediated retrograde signalling pathway with activation of AMPK and NF-κB, in which ROS induced mitochondrial membrane damage is a component of the signalling pathway [149,150]. Of note, the regulators involved in macrophage M2-like activation are also shared by the retrograde signalling pathway. Depletion of glucose or a glucose-rich hypoxic ROS environment favours M2-like activation or M1-like activation of macrophages, respectively and depletion of glucose can disarm T cells in the TME [132,151]. Low levels of ATP due to dietary restrictions or energy consumption, induce expression of nicotinamide phosphoribosyl transferase that generates NAD^+^, which is a key factor for SIRT1 activation. SIRT1, also a regulator of retrograde signalling, acetylates and activates PGC1ß to increase OXPHOS in macrophage [152,153,154].

In addition, Akt activation in response to mitochondrial respiratory stress has been found in different tumour cell systems [63,72,155] and PI3K/Akt signalling also regulates macrophage activation in the TME. The PI3K regulator lipid phosphatase, phosphatase and tensin homolog (PTEN), contributes to macrophage polarization because deletion of PTEN and resultant activation of PI3K/Akt signalling results in increased M2-like activation [156,157]. Interestingly, deletion of Akt1 promotes upregulation of inducible NO synthase and IL-12β (M1-like activation) and Akt2 deficiency in macrophages highlights the opposing roles of Akt isoforms in macrophage polarization, because Akt2−/− macrophages possess an M2-like phenotype [158]. These findings suggest that mitochondrial stress induced retrograde signalling can determine the activation of myeloid cells. Tumour suppressor p53 is critically important in preventing oncogenesis but its role in inflammation in general and in the function of inflammatory macrophages in particular is not certain. Mitochondrial stress induced by doxorubicin or partial depletion of mtDNA (~70%) activates the Ca2+/calcineurin retrograde signalling pathway, inducing expression of p53, which in turn attenuates HIF-1α activity in multiple types of cells [159,160,161]. p53 mediated suppression of HIF-1α controls regulation of glycolysis and ‘loss of function mutation’ of p53 is partially responsible for enhanced glycolysis in cancer cells [162]. Notably, deletion or activation of p53 in myeloid cells represses or induces the M2-like phenotype, respectively, in a chronic inflammatory venous thrombus model [163]. However, conflicting reports that p53 drives M1-like phenotype in tumour-associated macrophages (TAMs) of several cancer models [164,165,166] do not support this speculation, possibly indicating pathological differences in different microenvironments or in different model systems.

## 6. Conclusions

Many important questions about the involvement of retrograde signalling pathways in cancer biology, for example, the specific sensors of different types of mitochondrial stress and the cell and tissue specificity of the signalling responses, are yet to be explored. Importantly, with a list of small molecules and potential protein regulators involved in retrograde signalling, it appears that we still do not clearly understand the identity of specific signalling molecules that can trigger the retrograde signalling so far either in yeast or mammals. In addition, what determines whether retrograde signalling results in beneficial or maladaptive responses, its significance in determination of cancer progression and which cell type in the TME is mostly affected, should be identified. For now, relevant studies are largely limited by the difficulty of experimentally manipulating mtDNA in cancer cells and the lack of animal models with mtDNA mutations. It is not technically feasible at this moment to differentiate mtDNA abnormalities in host cells from those of the cancer cells in the TME. Would it be possible for mtDNA from cancer cells to move to and affect the non-transformed host stromal cells that have no genetic or epigenetic changes? Indeed, intercellular structures such as TNTs have been involved in the transfer of mitochondria between different cells. Thus, are such structures involved in the retrograde signalling and responsible for the communication between cells in the TME? Further studies on the horizontal transmission of mitochondrial retrograde signalling between diverse cells in the TME may also extend our understanding of cancer progression. As stated, retrograde signalling may suppress glycolysis and promote OXPHOS in myeloid cells, indicating a possibility that the retrograde signalling may regulate glycolysis in some type of cancer cells whose metabolism and genetic or epigenetic events are rewired. If it actually happens, this will also extend our understanding about functional implications of retrograde signalling. Finally, we expect the feasibility of future approaches, targeting retrograde signalling for successful cancer therapy from suppression of metastasis or resistance to drugs, to become an exciting topic of study in the near future.

## Figures and Tables

**Table 1:** Summary of oncometabolites.

Metabolites	Producer	Specific Function	Common Function
Acetyl CoA	PDC	Produces acetyl-CoA and increases histone acetylation. Increases the expression of genes that promote cell cycle progression and cell proliferation	
2-hydroxybutyrate	Mutated IDH	Inhibits cytochrome c oxidase and ATP synthase.	
Fumarate	WT SDH, mutated FH	Inhibits Keap1-mediated degradation of NRF. Increases ROS signalling via generation of succinated glutathione. Inhibits the demethylase KDM2B, increases H3 methylation and promotes binding of DNA-dependent protein kinase and the recruitment of end-processing DNA repair enzymes.	Inhibits the JMJD family, TET family and PHD family. Increased methylation promotes the expression of genes increasing proliferation and inhibiting differentiation.
Succinate	Mutated SDH		
Phosphoenolpyruvate (PEP)	PKM2	Phosphorylation of H3 with PEP facilitates H3 acetylation, promotes expression of c-Myc and cyclin D1. STAT3 phosphorylation promotes MEK5 activation.	

## Abbreviations

AICAR	5-Aminoimidazole-4-carboxamide ribonucleotide
AMPK	5′ AMP-activated protein kinase
ATFS-1	Activating transcription factor associated with stress-1
CAMKIV	Calcium/calmodulin-dependent protein kinase type IV
CREB	cAMP response element-binding protein
DOT1L	Disruptor of telomeric silencing 1-like
EMT	Epithelial to mesenchymal transition
ETC	Electron transfer chain
FAO	Fatty acid oxidation
HIF-1α	Hypoxia-inducible factor-1α
JNK	c-Jun N-terminal kinase
Keap1	Kelch-like ECH-associated protein 1
α-KG	α-Ketoglutarate
MAPK	Mitogen-activated protein kinas
MCT4	Monocarboxylate transporter 4
MMP-9	Matrix metalloproteinases-9
mtDNA	Mitochondrial DNA
nDNA	Nuclear DNA
NF-κB	Nuclear factor kappa-light-chain-enhancer of activated B cell
NFAT	Nuclear factor of activated T-cells
NO	Nitric oxide
NRF2	Nuclear factor erythroid 2-related factor 2
OXPHOS	Oxidative phosphorylation
PARP	Poly (ADP-ribose) polymerase
PGC1ß	Peroxisome proliferator-activated receptor gamma coactivator 1-alpha
PI3K	Phosphoinositide 3-kinase
PKC	Protein kinase C
ROS	Reactive oxygen species
SAPK	Stress-activated protein kinase
SIRT1	Silent mating type information regulation 2 homolog 1/Sirtuin
STAT3	Signal transducer and activator of transcription 3
TCA cycle	Tricarboxylic acid cycle/Krebs cycle
Treg	regulatory T cell
